# The psychosocial impact of receiving a polygenic risk score informing risk of prostate cancer

**DOI:** 10.1186/s13053-026-00347-w

**Published:** 2026-07-15

**Authors:** Matthew Hogben, Emma Hainsworth, Barbara Benton, Jana McHugh, Kathryn Myhill, Eva McGrowder, Reshma Rageevakumar, Denzil James, Sarah Benafif, Elizabeth C. Page, Rosalind A. Eeles, Emma Rowland, Elizabeth K. Bancroft

**Affiliations:** 1 Oncogenetics Research Team, The Royal Marsden NHS Foundation Trust, London, SW3 6JJ UK; 2 Oncogenetics Research Team, The Institute of Cancer Research, London, UK; 3 University College London Hospitals NHS Foundation Trust, London, UK; 4 Florence Nightingale Faculty of Nursing, Midwifery & Palliative Care, King’s College London, London, UK

**Keywords:** Risk communication, Genetic screening, Genetic testing, Polygenic risk score, Prostate cancer, Precision medicine, Psychosocial impact, Qualitative research

## Abstract

**Background:**

Use of polygenic risk scores (PRS) as a risk-stratification tool in cancer screening is an area of increasing interest. The BARCODE1 study is an observational prostate cancer screening study, investigating the use of PRS to risk-stratify people by risk of prostate cancer, with those identified as higher risk invited for prostate cancer screening. Participants of BARCODE1 were invited to take part in a psychosocial sub-study aiming to explore the effect of receiving a polygenic risk score on psychological health, family risk communication and impact on health behaviours.

**Methods:**

1300 participants of the BARCODE1 study were invited to complete questionnaires before and after receiving the results of their PRS. A sub-group were invited to participate in one-to-one in-depth semi-structured interviews, conducted in-person, by telephone or video-call. Interviews were audio recorded and transcribed verbatim. Data were analysed using Reflexive Thematic Analysis and the results are reported here.

**Results:**

Eighteen participants were interviewed and four themes identified; (a) mutual benefit, (b) emotional impact, (c) risk communication and (d) stoical attitudes to health. Participants reported minimal short-term impact on psychosocial health reported following receipt of a PRS. All participants communicated their genetic risk with their spouse/partner; however, some chose not to inform their children. Participants described themselves as having typical ‘male’ stoical responses to managing their health and that they did not feel the PRS results had an impact on their health behaviours.

**Conclusions:**

Providing an individualised PRS informing future risk of prostate cancer was well received. Overall, participants did not report being psychologically, socially or emotionally affected by receiving this genomic risk information. For those identified as being at higher risk it is important that there is access to healthcare professionals who are competent in discussing the meaning of the results.

**Trial registration:**

ClinicalTrials.gov NCT03857477, registered 26th Feb 2019.

**Supplementary Information:**

The online version contains supplementary material available at https://doi.org/10.1186/s13053-026-00347-w.

## Background

Prostate cancer (PrCa) is the most common cancer in people born with a prostate in the United Kingdom (UK), with 1 in 8 affected by age 80 [1]. For people of Black ethnicity this rises to 1 in 4 [1]. In 2023, 55,241 PrCas were diagnosed in England. This is a 25% increase from 2021, and 90% of diagnoses were in people of White ethnicity [2]. The prognosis for those diagnosed with early stage localized PrCa is good with the 5-year survival rate being almost 100% [1]. However, those with metastatic disease at diagnosis have a 5 year survival of 50% [1]. The UK National Health Service (NHS) Long Term Cancer plan has an ambitious aim of 75% of all cancer diagnoses being at an early stage by 2028 [3], and therefore innovative cancer screening methods are urgently required.

Risk factors for developing PrCa are age, ethnicity, a family history of PrCa or carrying a germline pathogenic variant in a PrCa susceptibility gene (for example *BRCA1*,* BRCA2*,* ATM*,* CHEK2*,* MLH1*,* MSH2* or *MSH6*) [4]. There is currently no recommended national PrCa screening programme in the UK. The prostate-specific-antigen (PSA) blood test is the most commonly used screening test, however, universal screening using a PSA test is not currently recommended due to the limitations of the test [1, 5]. Screening based on PSA can lead to the overdiagnosis and over treatment of low-grade cancers. Clinical practice has moved away from using digital rectal examination in the diagnostic pathway for prostate cancer (https://www.baus.org.uk/). Treatments for PrCa can have life-changing side-effects with high rates of erectile dysfunction and urinary incontinence reported post-surgery and radiotherapy [6]. Many people with small volume or low-grade tumours are managed using Active Surveillance, with regular PSA, MRI and biopsies, reverting to radical treatment if there is evidence of tumour progression [1].

PrCa has a large genetic component, with 58% heritability observed in studies of twins [7]. The genetic predisposition to PrCa is due to a combination of common and rare inherited constitutional (germline) genetic variants [4]. Rare variants (for example variants in the *BRCA1* and *BRCA2* genes) affect approximately 5–12% of people diagnosed with PrCa [8]. Therefore, whilst these rare variants have a role in the genetic predisposition to PrCa, they do not explain the majority of the familial risk [9].

Common genetic variants, or single nucleotide polymorphisms (SNPs), play an important contribution to the risk of PrCa. SNPs are single base changes in the DNA which each individually have a small impact on risk. Over 450 SNPs have been identified that affect PrCa risk [10]. A polygenic risk score (PRS) is calculated by multiplying the per allele odds ratio of the PrCa risk SNPs found in a person’s DNA, to give an individualised risk score [11, 12]. For people identified as having a polygenic risk score in the top decile their risk is estimated as being at least 3 times that of the general population risk. This is a similar level of risk that a person diagnosed with a pathogenic variant in the *BRCA2* gene. The BARCODE1 study evaluated how PRS can be used to stratify people from the general population aged between 55 and 69 years into different risk categories for targeted PrCa screening [13, 14]. The baseline results suggest that offering prostate screening to all participants in the top decile of the PRS detects clinically significant PrCa (defined as Gleason score *≥* 7) at an early stage, and further follow-up is ongoing. These results suggest that PRS could be used as part of routine PrCa risk-assessments in the coming years [14].

The vision of the NHS 10-year plan is to evaluate the use of PRS in targeted screening and prevention initiatives, and therefore research into the clinical implementation and patient experience of PRS is needed [15]. To date, there has been limited research looking at the psychosocial impact of being given PRS results to predict future risk of cancer [16–20]. Whilst testing pathways for high and moderate risk genes is well established, assessment of polygenic risk is a relatively new approach, and currently only available in a research setting or some private settings. Some studies using PRS within a PrCa setting have looked at the psychosocial impact of providing a PRS to people with a family history (FH) of PrCa. These studies have demonstrated how lived experience of PrCa in close relatives has had a powerful impact on how people interpret their personal risk [16, 20]. Understanding the psychosocial impact of introducing PRS as a new risk assessment tool in people without a family history of PrCa from the perspective of the service user is vital to assess psychological or social harms, such as increased anxiety or exacerbating inequalities, which would need to be addressed before widespread adoption.

To our knowledge, no research has reported the psychosocial impact of using a PRS in people in the general population to understand their future risk of PrCa and to offer targeted screening for them. The BARCODE1 study, described in further detail below and in McHugh et al., 2025 and Benafif et al., 2022 [13, 14], recruited cancer-free people to undergo a PRS, subsequently offering PrCa screening to those identified at higher-risk. The present sub-study ascertains the psychosocial impact of receiving a PRS for risk of PrCa. It aims to explore the effect of receiving a PRS on psychological health, family risk communication and impact on health behaviours.

## Methods

### The BARCODE1 study

The BARCODE1 study (ClinicalTrials.gov number, NCT03857477) recruited over 5000 people from the general population to undergo a genetic risk assessment for risk of PrCa. PrCa screening was offered to those identified in the top decile of the risk distribution. Initially a pilot study recruiting 300 participants from seven General Practices (GPs) was conducted from April 2016 to April 2018 to inform feasibility of the full study. For the full study, participants were recruited via 69 GP practices across the Southeast of England between March and July 2019. Collaborating GP practices screened their patient databases, and eligible individuals were invited by letter. Eligibility included people assigned male at birth, aged 55–69 years, of White European ethnicity (self-reported), without a personal history of PrCa or recent investigations for suspected PrCa. The rationale for inclusion criteria for limitation to people of White European ethnicity only is due to the PRS only being validated in people of this ethnicity at the time the study was conducted. Potential participants were sent an invitation letter, a health-screening questionnaire and a consent form. The health-screening questionnaire was reviewed by a study nurse to ensure eligibility against the criteria outlined above. Those deemed eligible were sent a postal saliva kit to provide a sample for genetic testing. PRS results were communicated by letter to participants within two years of enrolment, followed by a telephone call from a study nurse for those in the top decile, and considered to be at higher-risk to discuss the result and next steps. Those in the top decile were offered a consultation with a study nurse or doctor to discuss PrCa screening. Those consenting to screening then underwent a PSA test, an MRI and a prostate biopsy. Those participants not in the top decile were informed that they were not identified as being at the risk level for the study and at ‘population risk’, with signposting for a discussion with their GP if they had a family history of prostate or other cancers, or symptoms that they were concerned about.

### Psychosocial sub-study

Alongside the screening study, the first 1000 participants to consent to the full study and the 300 participants of the pilot study were invited to join a psychosocial sub-study, involving completion of surveys at study enrolment, after receiving their PRS results and at 12 months follow-up with a subset of 22 participants invited to take part in in-depth interviews after completing the final questionnaire. Participants were asked on the questionnaire about their willingness to be interviewed.

### Study design

Semi-structured interviews were conducted using an interview schedule developed by two researchers (MH and BB; see supplementary material). Interviews lasted between 20 and 50 min and were conducted either face-to-face in a London hospital, by video-call or by telephone, depending on participant preference. Interviews were audio recorded and transcribed verbatim by a professional transcribing company and returned transcripts were anonymised using pseudonyms and checked against the original recording for accuracy by the research team (MH, BB).

Purposive sampling was used to include participants with a variety of experiences within the study, to include those at different levels of risk, those with and without a subsequent cancer diagnosis following study investigations, those with and without a family history of PrCa, as well as varied sociodemographic factors such as job status, marital status, whether they had children or not and from across the age range of the study. The rationale for this sampling approach was to understand and explore the impact of receiving a PRS from multiple perspectives. There was no target sample size, instead was adequate sample size was determined by the information power criteria outlined by Malterud et al. (2016) [21]. Using this framework, the sample size was determined by the narrow study aim, the sample specificity (purposive sampling of participants holding characteristics that are highly specific for the aim), the high-quality of the data elicited from the in-depth interviews combined with the in-depth approach to the analysis.

### Data analysis

Data were analysed using Reflexive Thematic Analysis (RTA) [22, 23]. Transcripts were read and re-read line-by-line to observe patterns in the data and coded independently by one researcher (MH). Initial inductive (elicited from the data) and deductive (derived from theory/wider literature) codes were determined and verified by the team (MH, ER and EB). These codes were discussed iteratively, refined and definitive codes applied across all transcripts. The researchers grouped the codes into code families and then into themes, acknowledging and reflecting on the knowledge and experience drawn from clinical practice and previous research in this process [22, 23]. These themes were reviewed against the data, refined and agreed by the research team (MH, ER and EB) to produce the final themes. All names and potentially identifiable data have been removed from quotes to protect the participants identity.

## Results

In total, 18 participants were interviewed, two declined due to not having time and two did not respond to the invitation to be interviewed. Six interviews were conducted face-to-face, seven by video-call and five by telephone. Table 1 summarises the characteristics of the participants.

**Table 1:** Study participants characteristics

Participant number	Marital status	Children	Job status	Age (yrs)	Family History of prostate cancer in a first or second degree relative	Prostate cancer diagnosed through the study	Risk status based on PRS
1	Married	Y	Retired	55–60	N	Y	High
2	Married	Y	Retired	60–65	N	Y	High
3	Married	Y	Retired	55–60	N	N	High
4	Married	Y	Working	60–65	Y	N	High
5	Married	Y	Working	60–65	N	Y	High
6	Married	Y	Working	55–60	N	Y	High
7	Partner	Y	Retired	60–65	N	Y	High
8	Married	Y	Working	55–60	Y	N	High
9	Widowed	Y	Working	60–65	N	Y	High
10	Divorced	Y	Retired	65–70	N	Y	High
11	Married	N	Working	55–60	N	N	High
12	Married	Y	Retired	65–70	N	N	High
13	Partner	N	Working	60–65	N	N	High
14	Married	Y	Retired	60–65	Y	Y	High
15	Married	Y	Retired	65–70	N	N	Low
16	Partner	N	Working	60–65	N	N	Low
17	Married	Y	Retired	60–65	Y	N	Low
18	Married	Y	Working	55–60	Y	N	Low

Analysis identified four themes; (a) mutual benefit of research, (b) emotional impact, d) risk communication and e) stoical attitudes to health.

### Mutual benefit of research

All participants, regardless of their risk category, expressed the altruistic sentiment that their motivation to take part in this research was influenced by a desire to inform and advance knowledge to improve future care for others *“Principally*,* I like to advance the knowledge of medical science”* (participant 2, age 60–65). They all also expressed that their participation had a two-way benefit – to help further research for others, and also a personal benefit to access PrCa screening and to understand more about their personal risk of PrCa with the hope of an earlier diagnosis:

*…to be perfectly honest with not knowing if you’ve got prostate cancer*,* or seemingly quite difficult to diagnose from blood tests I was receiving*,* I willingly participated. It’s only going to benefit me personally in the long run and maybe help other people as well. (Participant 4*,* 60–65)*

This was true of participants who had a family history of PrCa, and also those who had no known family history. The participants expressed that by contributing to medical research then their hope was the routine use of PRS could lead to earlier diagnosis of treatable disease.

*my reason for joining was the hope this test might lead to better ways of catching prostate cancer early*,* I’ve seen in my friends how late*,* how devastating catching it late can be* (participant 17, age 60–65)

Participants described PrCa as a considerable concern for men and therefore they embraced the opportunity to have a genetic test to have more information about their personal risk and to use that as a means of accessing intensive screening tests. For some they also described the hope that this would also provide reassurance about being ‘clear’ of PrCa.

*I thought*,* ‘That sounds like a lot more bangs for bucks.” I will not just get a PSA test; I will get a load of other interesting things. So*,* it will give me a clearance on one area.* (participant 3, *age 55–60*)

### Emotional impact

Three participants described the receipt of their PRS result, placing them in a high-risk group as provoking a strong response of feeling *“shocked”* and *“emotionally impacted”* by the result. Two participants (participant 5 age 60–65) describe strong reactions of shock in receiving their PRS, *“Oh*,* you mean the “oh*,* shit” moment”* and another *“There is an evolution*,* so I will start at the beginning. Four letters*,* word begins with F and ends in K”* (participant *3*, age 55–60). Another describes a *“temporary anxiety”* upon receiving his PRS (participant 6, age 55–60). Participants in the high-risk group described the importance of having a call from a Genetics Nurse within a few days of receiving their results and knowing they had a contact point for any questions:

*Knowing someone was going to call me was a big relief*,* so I had the chance to process the information and think about (questions) and what it all meant for me and my family* (participant 9, age 60–65)

All other participants receiving a higher risk result did not recall a significant emotional impact when receiving their result. Two participants described *“surprise”* at their result, mainly due to not having a family history of PrCa, which was an underlying assumption described by many participants.

*I probably misinterpreted it at the time*,* I thought it was based on my family history. … It really didn’t worry me because probably at the time I didn’t really understand it. (Participant 14*,* age 60–65 years)*

Others recalled receiving the result, but described receiving the result as of interest rather than having an impact on them emotionally, and none of the participants described needing any additional psychological support, summarised by participant 11:

*Finding out that I was in the top 10% of the people that would more or less get it*, (did it affect me?) *no*,* not really. It doesn’t mean you are going to get it’.’ I don’t think I needed any support. I mean there are some fragile people these days but I’m not one of them* (participant 11, age 55–60)

None of the participants in the population risk group could recall how they felt emotionally when they received their result, expressing that they felt it had minimal impact on them.

### Risk communication

This theme captured the pattern of participant’s discussions around how they talked about their PRS results and disclosure of their risk status to those close to them, and their wider social circle. In discussing who they shared their PRS result with, all participants in a partnership disclosed the results to their spouses/partners as they described sharing all health information with them. Often they explained they had been part of their decision-making to join the study. Although participant 12 (age 65–70) who had a higher risk result chose not to disclose his results until after he had undergone all screening investigations. He rationalised this decision by saying that if he was diagnosed with PrCa he did not want to be treated as *“someone with an illness”*, he did not want to be treated differently because of this genetic risk information. However, when he received his negative prostate biopsy result, he disclosed everything to his wife and children.

*I decided for a little while to keep it to myself because I’ve got two daughters and a wife*,* and I’m afraid I can deal with the being unwell*,* but I can’t deal with people faffing around you. And now you’re ‘David with an illness’*,* not David.* (participant 12 age 65–70 (name changed).

This desire to manage the concern raised by a high-risk result was also seen in communication with adult children, whilst most participants disclosed their results to adult children, four participants chose not to disclose their results to their children immediately after finding out, justifying this by saying they did not want to worry them:

*Latterly I did tell my children*,* and they were a little bit concerned by me not informing them. At the time there wasn’t any evidence to suggest that there was anything wrong anyway. (participant 4*,* age 60–65).*

For those with a population risk result they reported telling their family but did not share more widely with their children or within their social circle. They described receiving the result and not giving it much thought beyond some initial relief that they didn’t need any additional investigations. *“I thought ‘phew’ I’m glad I don’t need to think about having to have extra tests*,* and then I can’t say I have thought about it since”*. (participant 15, age 65–70)

Only five participants with a high-risk result discussed their PRS result with their wider social circle, including friends and colleagues. The other participants described how they preferred to keep things to themselves and not discuss them outside of their family unit. Those that did disclose more widely described using their experience of the research project and PRS result as an opportunity to raise awareness about PrCa in those close to them. Two participants described speaking with work colleagues who are a similar age to encourage them to seek PSA screening. Similarly, one participant discussed his cancer diagnosis with work colleagues who also had also been diagnosed with cancer, finding support from their shared experiences.

*We (colleagues) talk openly. Those of us that are in the club*,* there is no embarrassment because we all know what it means or what it could mean in the future…. a good support network*,* you start comparing war stories*,* right? (participant 13*,* age 60–65)*

### Stoical attitudes to health

The participants interviewed were very stoical about their approach to their health and consequent health behaviours, describing themselves as ‘typical men’, with a tendency to play things down and not liking any fuss about their health:

*I’m a typical man, just bottle things up really (participant 1, age 55–60)*

Participants described themselves as not emotional, not dwelling on things and having more of a practical response to risk, identifying this as a *‘stoical’* stance. This was seen in their description of ‘just getting on’ with the PrCa screening that was advised, or conversely for those at population risk just accepting their result and moving on.

*I tend to be one of these people who accepts things in life and gets on with it’*

*Just got on and dealt with it. (participant 7, age 60–65)*

As seen with their descriptions of telling others about their results, several participants expressed a practical need to deal with it on their own, to get on with having prostate screening, and not involve others in the process:

*‘I’ll be glum on my own. I just crack on with it. When I go and do a blood test*,* in*,* out*,* done and away. I don’t involve anyone in any of it.’ I tend to not be a feelings person. I don’t want to go and talk to a load of old men about their prostate. (participant 10*,* age 65–70)*

None of the participants interviewed reported that receiving their PRS resulted in them altering their lifestyle or health behaviours, for example changing their diet or exercise regime or feeling more concerned about potential symptoms.

*I would honestly say no because a lot of this is*,* “What can I do about it?” It’s not exactly anything that you can change your lifestyle with*,* well as far as I know. (participant 8*,* age 55–60)*

Four participants were diagnosed with other health conditions, for example diabetes during their study involvement which they reported had a greater impact on their desire to change their diets and health behaviours.

*lifestyle changes have just been more to keep myself getting away from being pre-diabetic.* (participant 13, age 60–65)

The participants receiving population risk results also didn’t report any changes in how they viewed their health or impact on health behaviours. They described understanding that they could still develop PrCa despite this result, and that they would follow their GPs advice:

*I know I could still get prostate cancer*,* I am generally quite good at seeing my GP if I have symptoms that are worrying me*,* whereas a I know a lot of men tend to avoid doing that (participant 18*,* age 55–60)*

There was nothing to suggest that receiving a PRS had any impact on the health behaviours of participants taking part in this study, either positively or negatively.

## Discussion

This study has explored the effect of receiving a PRS within the BARCODE1 study on the participants in terms of their psychological health, family risk communication and impact on subsequent health behaviours. We found that the participants reported a mutual benefit in seeking out the research in terms of helping to advance research, as well as finding out more about their own personal risk, that there was a minimal emotional impact for the majority and that most participants told their family about their PRS results, although some withheld the information from adult children. Participants described themselves as having typical ‘male’ stoical responses to managing their health and that they did not feel the PRS results had an impact on their health behaviours.

In terms of motivation to take part in research, the ‘mutual benefit’ of finding out about one’s personal risk as well as furthering research has been reported in other studies [20, 24, 25]. This is perhaps particularly pertinent in PrCa where there is no general population screening programme in place, and advice about PrCa is sought through shared-decision making with a primary care provider. It is well understood that a family history of PrCa is a risk factor for developing the disease [4], and this awareness was apparent within those interviewed with some participants reporting that this influenced their decision to join the research.

There was no evidence of a negative impact on participant’s medium and longer-term psychosocial functioning, nor their emotional wellbeing as a consequence of receiving their PRS within this study. A small number of participants described a strong initial emotional reaction to receiving a high PRS result, describing this as an initial shock, but this soon passed with no longer lasting impact recalled. This matches the findings of previous research that has looked at providing PRS to people with a family history of PrCa [16], and a personal or family history of breast cancer [19]. However, in contrast to this study, both of these studies provided participants with genetic counselling due to their personal or family history of cancer prior to receiving their PRS. The role of genetic counselling on the emotional reaction to receiving PRS is unknown. It is reassuring to see that despite this difference in approach and lived experience, the PRS was well received and did not appear to have a negative impact on the psychological health of the participants. Similar findings are also reported in people receiving results of testing for rare genetic variants conferring a very high-risk of cancer [25]. This provides further evidence that receiving genetic test results for cancer risk does not cause long-term psychological harm for the recipient across different risk settings.

We found that participants in our study shared their results with those closest to them but were less likely to share their experience with their wider family and social circle. There are known to be a number of potential barriers to genetic risk communication in families with high-risk genetic variants for example poor family relationships and not being in regular communication with relatives leading to challenges in alerting others in the family to a potential genetic predisposition [26, 27]. However, PRS does not have the same implications for blood relatives as having a dominantly inherited high-risk genetic variant (such as in the *BRCA1* or *BRCA2* genes), and so this more personal result tended to be kept more private. When the result was shared more widely it was used as a method of communicating general PrCa risk to other people to encourage them to seek PrCa screening. Similar findings were identified by Bancroft et al. [16] where men saw themselves as ‘risk communicators’ with a responsibility of raising awareness of PrCa amongst their peers.

The study participants described a ‘typically male’ stoical approach to their health, tending to play things down and not wanting to make a fuss about their health, which has been previously described within health screening, cancer treatment and genetic research [16, 28, 29]. We know from the work of others that perceptions of masculine behaviours can impact on the way that people interact with health services, with attributes such as self-reliance and avoidance of femininity acting as potential barriers to screening. It is important that healthcare professionals are aware of how these attitudes to health can impact on the uptake of cancer screening, delay help-seeking and lead to underreporting of symptoms [30]. Whilst the participants of this study had overcome this to proactively join this research these embedded masculine qualities may have led them to underplay the emotional impact of receiving the PRS [29].

It was reassuring to see that the PRS results did not have an adverse impact on the participant’s reported health behaviours. For example, those at higher risk did not report that this made them more concerned about looking out for potential symptoms of prostate cancer. In addition, participants who had a population risk PRS result were able to correctly identify that they could still develop PrCa and would not ignore symptoms on the basis of the PRS result. Other studies exploring the provision of PRS results for risk of breast and colorectal cancer found similar results, with no change to health behaviours reported [17, 24, 31]. However, one study did find that some participants given PRS for risk of colorectal cancer had a positive impact on making changes to their diet and exercise regimes based on a high PRS [29]. However, there is stronger evidence for the role of dietary changes in reducing the risk of colorectal cancer compared with PrCa.

### Clinical implications and future directions

This study has described the experience of being given a PRS to determine future risk of PrCa. The results are reassuring that the majority of participants did not experience any adverse psychosocial impact nor do they report any negative impact on health behaviours. This is important information for clinicians to be aware of as PRS becomes routinely implemented into standard care pathways. It will be important for future research to focus on the longer-term impact of living with results of a PRS. Whilst this study confirms the findings of others that there is minimal impact on psychological health or health behaviours in the short-term, there is limited knowledge about how this may change over time in this general population sample and the optimal information and support needed alongside testing needs to be determined. The needs of this study population may differ from the context of living at higher risk due to a genetic predisposition and/or family history [17, 24]. It is important for research to explore whether there has been any impact of providing PRS on other services, for example whether those told they have a higher risk of cancer increase their interactions with health care providers with concern about potential symptoms of cancer.

### Strengths and limitations

A strength of this study is that it included a range of participants perspectives by including both those with and without a diagnosis of PrCa (identified through screening within the study), and with a range of PRS results. However, due to the design of the BARCODE1 study all participants were limited to those of White European ethnicity and aged between 55 and 69 years [14]. The participants were from a similar social backgrounds which may limit the knowledge gained from this study when considering PRS in different setting and with different populations. If PRS is to be offered as a future method of risk-stratifying people from the general population then it is vital that future research is designed to include participants that are more representative of the UK population, including those from diverse sociodemographic and ethnic backgrounds as well as gender diversity. This is particularly important for people of Black ethnicity due to their increased risk of PrCa compared with those of White or Asian ancestries.

## Conclusions

Providing an individualised PRS informing future risk of PrCa was well received amongst study participants. We did not detect any long-term adverse impact on psychological, social or emotional health in those receiving a personalised PRS. For those identified as being at higher risk it is important that there is access to healthcare professionals who are competent in discussing the meaning of the results and future research should examine the longer-term outcomes and impact on health behaviours and in a more diverse study population.

## Supplementary Information

Below is the link to the electronic supplementary material.

Supplementary Material 1

## Data Availability

Anonymised datasets used and/or analysed during the current study are available from the corresponding author on reasonable request.
