# Supplementary material for: The psychosocial impact of receiving a polygenic risk score informing risk of prostate cancer
Source: Hered Cancer Clin Pract. 2026 Jul 15;24:19. doi: 10.1186/s13053-026-00347-w (PMC13656678; doi:10.1186/s13053-026-00347-w)
Supplement: Supplementary file 1 [file 13053_2026_347_MOESM1_ESM.pdf]

ICR

The ROYAL MARSDEN
NHS Foundation Trust

Supplementary Document: Interview Schedule

The BARCODE 1 Study: A psychosocial sub-study investigating the information requirements of men undergoing genetic-risk profiling

Interview Schedule

(Not intended to be structured, but areas to be covered during the interview).

1. Tell me about your understanding of the role of genetics in the development of prostate cancer.
2. What influenced you to decide to take part in the BARCODE 1 study?
3. Did you have any concerns about taking part in the study?
4. What is your impression about your risk of prostate cancer? How has this changed through taking part in this research?
5. Do you think that taking part in the study has had an impact on your anxiety/worry about prostate cancer?
6. Talk me through your genetic test results. What is your interpretation of the genetic information that you were given?
7. Has the information you were given about the study prepared you for the results you received?
8. Were you given sufficient time to discuss what being in the top 10% meant?
9. Was there any additional information or support you would have liked both before and after receiving these results?
10. Did you discuss your high polygenic risk score result with anyone? If so, who? It not, can you tell me why?
11. How do you feel about the future development of a standard genetic test for prostate cancer predisposition?
12. If there was such a test, what would your attitude be about men in your family taking it?
13. How much trust would you have in a test?
14. When genetic testing for prostate cancer becomes available in the future how do you suggest we (healthcare providers) give this information? What support should be provided along side this?
15. Do you think this information should be available to anyone other than the man having the test (such as his GP / family members)?
16. Has taking part in this study changed your intention to access screening for prostate cancer now / in the future?
17. How do you think that such information may impact on future healthcare (in terms of screening, decision-making etc)?
18. What do you think would influence uptake of such a test?
